# Brodalumab for the treatment of plaque psoriasis in a real-life setting: a 3 years multicenter retrospective study—IL PSO (Italian landscape psoriasis)

**DOI:** 10.3389/fmed.2023.1196966

**Published:** 2023-07-03

**Authors:** Luigi Gargiulo, Luciano Ibba, Piergiorgio Malagoli, Fabrizio Amoruso, Giuseppe Argenziano, Anna Balato, Federico Bardazzi, Martina Burlando, Carlo Giovanni Carrera, Giovanni Damiani, Paolo Dapavo, Valentina Dini, Gabriella Fabbrocini, Chiara Franchi, Francesca Maria Gaiani, Giampiero Girolomoni, Claudio Guarneri, Claudia Lasagni, Francesco Loconsole, Angelo Valerio Marzano, Matteo Megna, Francesca Sampogna, Massimo Travaglini, Antonio Costanzo, Alessandra Narcisi

**Affiliations:** ^1^Dermatology Unit, IRCCS Humanitas Research Hospital, Milan, Italy; ^2^Department of Biomedical Sciences, Humanitas University, Milan, Italy; ^3^Dermatology Unit, Department of Dermatology, Azienda Ospedaliera San Donato Milanese, Milan, Italy; ^4^Dermatology Unit, Azienda Ospedaliera di Cosenza, Cosenza, Italy; ^5^Dermatology Unit, University of Campania L. Vanvitelli, Naples, Italy; ^6^Dermatology Unit, IRCCS Azienda Ospedaliero-Universitaria di Bologna, Policlinico S. Orsola Malpighi, Bologna, Italy; ^7^Department of Dermatology, Dipartimento di Scienze della Salute (DISSAL), University of Genoa, IRCCS Ospedale Policlinico San Martino, Genoa, Italy; ^8^Dermatology Unit, Fondazione IRCCS Ca’ Granda Ospedale Maggiore Policlinico, Milan, Italy; ^9^Department of Biomedical, Surgical, and Dental Sciences, University of Milan, Milan, Italy; ^10^Department of Biomedical Science and Human Oncology, Second Dermatologic Clinic, University of Turin, Turin, Italy; ^11^Dermatology Unit, Department of Clinical and Experimental Medicine, Ospedale Santa Chiara, Pisa, Italy; ^12^Section of Dermatology, Department of Clinical Medicine and Surgery, University of Naples Federico II, Naples, Italy; ^13^Department of Medicine, Section of Dermatology and Venereology, University of Verona, Verona, Italy; ^14^Unit of Dermatology, Department of Biomedical and Dental Sciences and Morphofunctional Imaging, University of Messina, AOU Policlinico G. Martino, Messina, Italy; ^15^Department of Specialized Medicine, Dermatological Clinic, University of Modena, Modena, Italy; ^16^Department of Dermatology, University of Bari, Bari, Italy; ^17^Department of Pathophysiology and Transplantation, Università degli Studi di Milano, Milan, Italy; ^18^Clinical Epidemiology Unit, Istituto Dermopatico dell’Immacolata (IDI) IRCCS, Rome, Italy; ^19^U.O.S.D. dermatologica—centro per la cura della psoriasi, Ospedale Perrino, Brindisi, Italy

**Keywords:** brodalumab, psoriasis, psoriasis treatment, psoriatic arthritis, real-life

## Abstract

**Introduction:**

Brodalumab is a monoclonal antibody that targets the subunit A of the interleukin-17A receptor (IL17RA), inhibiting the signaling of various isoforms of the IL-17 family. It has been approved for the treatment of moderate-to-severe plaque psoriasis after being evaluated in three Phase-3 trials. However, long-term data on brodalumab in a real-life setting are still limited.

**Methods:**

The aim of this study was to evaluate the long-term effectiveness and safety of brodalumab in psoriasis. We also assessed the drug survival of brodalumab in a 3 years timespan. We conducted a retrospective multicenter study on 606 patients followed up at 14 Italian dermatology units, all treated with brodalumab according to Italian guidelines. Patients’ demographics and disease characteristics were retrieved from electronic databases. At baseline and weeks 12, 24, 52, 104 and 156, we evaluated the psoriasis area and severity index (PASI) score and investigated for adverse events. The proportions of patients reaching 75, 90 and 100% (PASI 75, PASI 90 and PASI 100, respectively) improvement in PASI, compared with baseline, were also recorded.

**Results:**

At week 12, 63.53% of the patients reached PASI 90 and 49.17% PASI 100. After 3 years of treatment, 65.22% of patients maintained a complete skin clearance, and 91.30% had an absolute PASI of 2 or less. Patients naïve to biological therapies had better clinical responses at weeks 12, 24 and 52. However, after 2 years of treatment, no significant differences were observed. Body mass index did not interfere with the effectiveness of brodalumab throughout the study. No new safety findings were recorded. After 36 months, 85.64% of our patients were still on treatment with brodalumab.

**Conclusion:**

Our data confirm the effectiveness and the safety of brodalumab in the largest real-life cohort to date, up to 156 weeks.

## 1. Introduction

Biological therapies represent the most promising treatment options for moderate-to-severe plaque psoriasis and psoriatic arthritis (PsA) (1, 2). Several drugs have been approved over the last 10 years, targeting the interleukin (IL)-23/17 axis, which has been established as the pivotal pathway in psoriasis pathogenesis (3–5). In particular, the family of IL-17 is composed of six members (from IL-17A to IL-17F) (6). In plaque psoriasis, the expression of IL-17A, IL-17F and IL-17C is strongly increased (7).

Three different mechanisms have been developed to target IL-17: two drugs (secukinumab and ixekizumab) inhibit specifically the IL-17A isoform (8, 9), bimekizumab blocks both IL-17A and IL-17F (10), while brodalumab is the only approved monoclonal antibody that selectively targets the subunit A of the IL-17A receptor (IL-17RA) (11). Since IL-17RA is involved in the signaling of several forms of the IL-17 family, brodalumab blocks the effects of IL-17A, IL-17F, IL-17E, and IL-17C (11).

Brodalumab is a fully human IgG2k monoclonal antibody, which has been approved to treat moderate-to-severe plaque psoriasis after being evaluated in three phase-3 clinical trials (AMAGINE-1, AMAGINE-2, and AMAGINE-3) (12, 13), showing superior efficacy compared with both placebo and ustekinumab. Brodalumab is administered with a single subcutaneous injection of 210 mg at weeks 0, 1, and 2 and then every 2 weeks (11).

Despite several recent published real-life experiences on brodalumab, only limited data are available regarding this drug’s long-term effectiveness and safety in a real-world setting (14–16). We conducted a multicenter real-life retrospective study to assess the effectiveness of brodalumab in patients treated for 156 weeks, also evaluating the drug survival and the safety profile.

## 2. Materials and methods

### 2.1. Study design

This is a real-life retrospective study conducted by analyzing the electronic databases of 14 Italian dermatology units. Six hundred and six consecutive patients were enrolled, all treated with brodalumab for moderate-to-severe plaque psoriasis for at least 12 weeks between June 2018 and January 2023. All patients received brodalumab in accordance with the Italian guidelines: at baseline, they had either a psoriasis area and severity index (PASI) ≥10 or a PASI <10 with the involvement of sensitive areas (including face, nails, palms/soles or genitals). All patients had previously received at least one conventional systemic drug or had a contraindication to those treatments. Before starting brodalumab, complete blood examinations were performed, including screening for hepatitis B, hepatitis C, HIV and tuberculosis (17). Brodalumab was administered according to the approved dosage of European Medicines Agency (EMA), and no frequency or dose modifications were allowed (11).

The characteristics of all patients, including age, comorbidities, disease duration, previous treatments and PASI scores at each visit, were obtained from electronic medical records. At weeks 12, 24, 52, 104 and 156, the proportions of patients reaching a reduction of 75%, 90%, and 100% in PASI compared with baseline (PASI 75, PASI 90 and PASI 100, respectively) were recorded. We also analyzed the percentages of patients who achieved an absolute PASI of 2 or less at each visit, following the Italian adaptation of EuroGuiDerm guidelines (18).

During each dermatological examination, patients were questioned about the onset of any adverse event (AE), including AEs leading to the discontinuation of brodalumab.

Given the retrospective design of our study, not all visits were completed by all patients. Therefore, all data for follow-up visits they had yet to attend were deemed missing.

Institutional review board approval was exempted, as the study procedures did not deviate from standard clinical practice. All included patients had provided written informed consent for the retrospective analysis of their clinical data.

### 2.2. Statistical analyses

Continuous variables were reported using mean and standard deviation (SD), while categorical variables were presented as absolute numbers and frequencies. The effectiveness of brodalumab in terms of PASI ≤2, PASI 75, PASI 90 and PASI 100 was evaluated according to different variables, including BMI, comorbidities and previous exposure to biological treatments. Chi-square test and exact Fisher’s test were used to analyze categorical variables, while student’s *t*-test and Mann–Whitney *U* test were used for continuous data. To test the differences between more than two groups, we used one-way ANOVA or Kruskal–Wallis test if the distribution was not normal. Multivariate logistic regression analysis was applied to assess the impact of all the already mentioned variables on the dependent variables PASI75/PASI90/PASI100 and PASI ≤2. We included all variables with a probability value (*p*-value) of less than 0.2 in the univariate analysis. Odds ratios (ORs) and 95% confidence intervals (CIs) were reported. All patients treated with brodalumab were included in the drug survival analysis using the Kaplan–Meier curves. The event date was chosen as the date the patient discontinued the drug by any cause. Time data was censored for patients still on treatment when the study was conducted and for patients lost to follow-up. The differences in drug survival between subgroups were assessed with the log-rank test. We also conducted Cox regression analysis to determine predictive variables for drug survival.

A *p*-value <0.05 was considered significant.

Microsoft Excel and STATA/SE 17.0 software were used to generate tables and graphs and for statistical analyses, respectively.

## 3. Results

### 3.1. Patients’ characteristics

Six hundred and six patients were included in our study. Five hundred and eighty-nine patients completed 24 weeks of treatment, while 553, 304 and 115 of them reached one, two and three years of follow-up, respectively. Four hundred and eleven were males (67.82%), the mean age was 52.55 years, with a SD of 14.90. Our patients were affected by plaque psoriasis for a mean of 19.44 years (SD 13.98). Mean body mass index (BMI) was 26.65 (SD 4.74), and 18.15% of our patients were obese, with a BMI ≥30. Previous diagnosis of psoriatic arthritis (PsA) was observed in 21.45% of patients, and at least one cardio-metabolic comorbidity (including obesity, arterial hypertension, type II diabetes mellitus, hypercholesterolemia and cardiovascular diseases) affected 50.33% of them. More than half of patients had previously received at least one biological drug (50.99%), with the most prescribed drug being adalimumab (20.63%), followed by secukinumab (15.51%) and etanercept (14.36%). Additional data regarding the characteristics of our populations are summarized in Table 1.

**Table 1:** Characteristics of the 606 patients at baseline.

Number of patients	606
**Mean (SD)**	
Age (years)	52.55 (14.90)
BMI	26.65 (4.74)
mPASI at baseline	15.44 (7.28)
Disease duration (years)	19.44 (13.98)
***N* (%)**	
Male	411 (67.82)
Obese (BMI ≥30)	110 (18.15)
PsA	130 (21.45)
Difficult-site involvement	430 (70.96)
Comorbidity	326 (53.80)
Cardiometabolic comorbidities	305 (50.33)
Hepatitis C	4 (0.66)
Hepatitis B	5 (0.83)
Bio-experienced	309 (50.99)
**Previous biologics**	
Adalimumab	125 (20.63)
Etanercept	87 (14.37)
Infliximab	10 (1.65)
Apremilast	14 (2.31)
Secukinumab	94 (15.51)
Ixekizumab	36 (5.94)
Ustekinumab	47 (7.76)
Guselkumab	15 (2.48)
Risankizumab	10 (1.65)
Tildrakizumab	7 (1.16)

SD, standard deviation; BMI: body mass index; PsA: psoriatic arthritis; PASI: psoriasis area and severity index; mPASI: mean PASI.

### 3.2. Effectiveness of brodalumab

**Figure 1:**
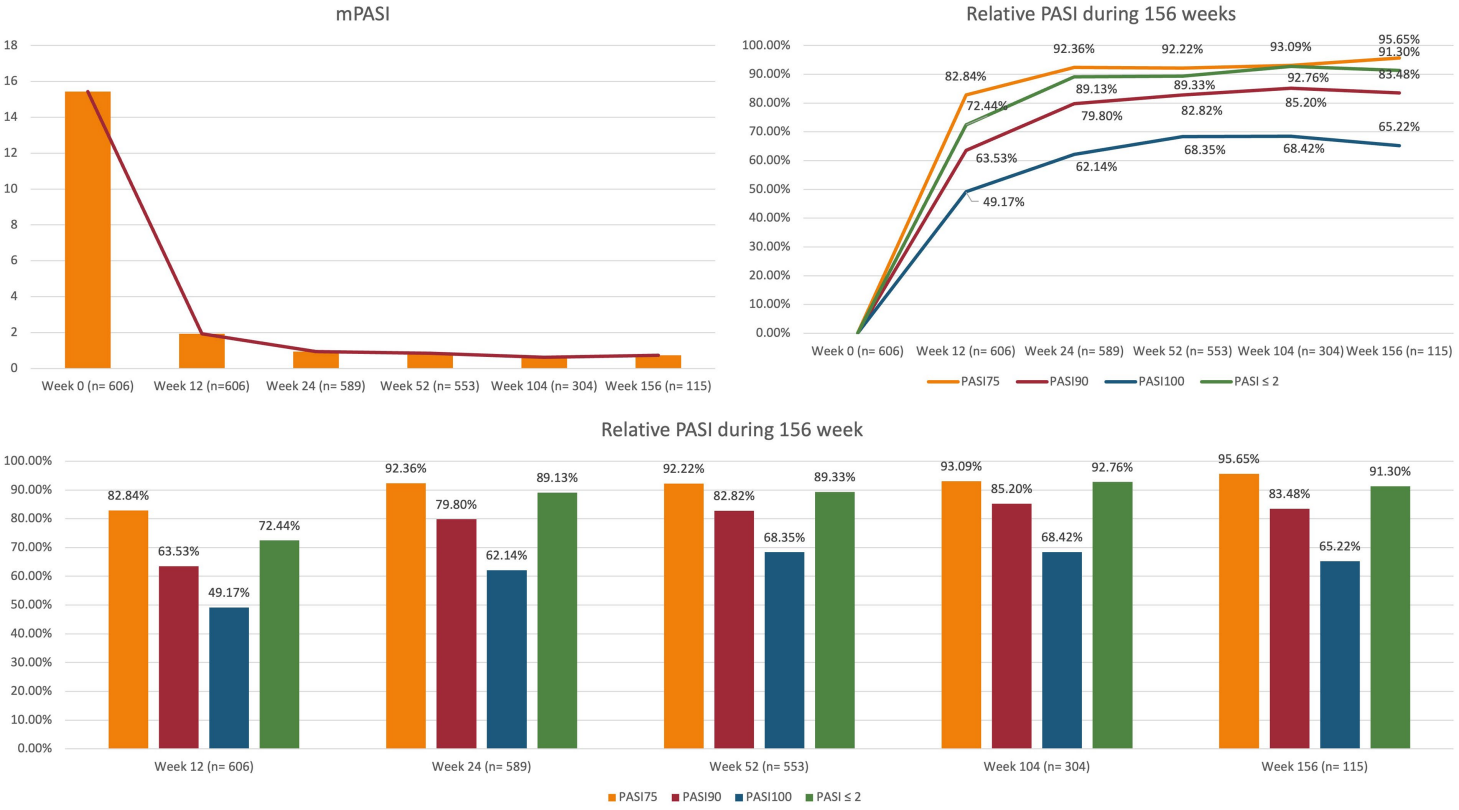
Clinical effectiveness of brodalumab throughout 156 weeks. PASI, psoriasis area and severity index; mPASI: mean PASI.

At baseline, the mean PASI was 15.44 (SD 7.28). During the treatment with brodalumab, it decreased to 1.93 (3.48) at week 12, 0.94 (1.88) at week 24 and 0.63 (1.99) after one year of treatment. Mean PASI was similar after two and three years of therapy, being 0.63 (1.30) and 0.74 (1.46), respectively. At week 12, 82.84% of the patients reached PASI 75, 63.53% PASI 90, 49.17% PASI 100 and 72.44% PASI ≤2. The effectiveness of brodalumab was maintained throughout the study period, with 82.82%, 85.20% and 83.48% of patients reaching PASI 90 after one, two and three years of treatment, respectively. After 156 weeks, 65.22% of patients maintained a complete skin clearance, and 91.30% had an absolute PASI ≤2. Additional data on the effectiveness of brodalumab at each time point in terms of mean PASI, PASI 75/90/100 and PASI ≤2 are shown in Figure 1.

### 3.3. Effectiveness of brodalumab in selected sub-populations

The effectiveness of brodalumab was analyzed in relation to several variables, including previous treatment with other biological drugs, presence of concomitant PsA, BMI classes, involvement of difficult-to-treat areas and diagnosis of at least one cardio-metabolic disease (CMD)arter. Data on the analysis of all subgroups are summarized in Tables 2, 3.

**Table 2:** Effectiveness of brodalumab according to previous exposure to biological drugs, concomitant psoriatic arthritis, presence of cardio-metabolic comorbidities and involvement of difficult-to-treat areas.

	Bio-naïve	Bio-experienced	Univariate analysis *p*-value	Multivariate analysis OR (CI 95%); *p*-value	PsA	No PsA	Univariate analysis *p*-value	Multivariate analysis OR (CI 95%); *p*-value
PASI75 w12	259/297 (87.21%)	243/309 (78.64%)	**0.005**	1.85 (1.20–2.86); 0.006	105/130 (80.77%)	397/476 (83.40%)	0.480	NA
PASI90 w12	212/297 (71.38%)	173/309 (55.99%)	**<0.001**	1.89 (1.34–2.65); <0.001	76/130 (58.46%)	309/476 (64.92%)	0.175	NS
PASI100 w12	165/297 (55.56%)	133/309 (43.04%)	**0.002**	1.55 (1.12–2.16); 0.008	53/130 (40.77%)	245/476 (51.47%)	**0.031**	NS
PASI ≤2 w12	237/297 (79.80%)	202/309 (65.37%)	**<0.001**	2.03 (1.40–2.94); <0.001	86/130 (66.15%)	353/476 (74.16%)	0.070	NS
PASI75 w24	272/288 (94.44%)	272/301 (90.37%)	0.062	NS	116/127 (91.34%)	428/462 (92.64%)	0.625	NA
PASI90 w24	252/288 (87.50%)	218/301 (72.43%)	**<0.001**	2.65 (1.72–4.1); <0.001	99/127 (77.95%)	371/462 (80.30%)	0.559	NA
PASI100 w24	200/288 (69.44%)	166/301 (51.15%)	**<0.001**	1.79 (1.27–2.52); 0.001	71/127 (55.91%)	295/462 (63.85%)	0.102	NS
PASI ≤2 w24	263/288 (91.32%)	262/301 (87.04%)	0.096	NS	114/127 (89.76%)	411/462 (88.96%)	0.797	NA
PASI75 w52	261/277 (94.22%)	249/276 (90.22%)	0.079	NS	112/118 (94.92%)	398/435 (91.49%)	0.218	NA
PASI90 w52	242/277 (87.36%)	216/276 (78.26%)	**0.005**	1.91 (1.21–3.01); 0.006	99/118 (83.90%)	359/435 (82.53%)	0.726	NA
PASI100 w52	202/277 (72.92%)	176/276 (63.77%)	**0.021**	1.50 (1.04–2.16); 0.029	77/118 (65.25%)	301/435 (69.20%)	0.414	NA
PASI ≤2 w52	250/277 (90.25%)	244/276 (88.41%)	0.842	NA	109/118 (92.37%)	385/435 (88.51%)	0.227	NA
PASI75 w104	139/147 (94.56%)	144/157 (91.72%)	0.329	NA	73/75 (97.33%)	210/229 (91.70%)	0.095	NS
PASI90 w104	134/147 (91.16%)	125/157 (79.62%)	**0.005**	2.61 (1.31–5.21); 0.006	67/75 (89.33%)	192/229 (83.84%)	0.245	NA
PASI100 w104	104/147 (70.75%)	104/157 (66.24%)	0.398	NA	47/75 (62.67%)	161/229 (70.31%)	0.217	NA
PASI ≤2 w104	139/147 (94.56%)	143/157 (91.08%)	0.243	NA	74/75 (98.67%)	208/229 (90.83%)	**0.023**	NS
PASI75 w156	55/58 (94.83%)	55/57 (96.49%)	0.662	NA	30/31 (96.77%)	80/84 (95.24%)	0.720	NA
PASI90 w156	46/58 (79.31%)	50/57 (87.72%)	0.225	NA	27/31 (87.10%)	69/84 (82.14%)	0.526	NA
PASI100 w156	35/58 (60.34%)	40/57 (70.18%)	0.268	NA	20/31 (64.52%)	55/84 (65.48%)	0.924	NA
PASI ≤2 w156	52/58 (89.66%)	53/57 (92.98%)	0.527	NA	30/31 (96.77%)	75/84 (89.29%)	0.206	NA

	CMD	No CMD	Univariate analysis *p*-value	Multivariate analysis OR (CI 95%); *p*-value	Difficult areas	No difficult areas	Univariate analysis *p*-value	Multivariate analysis OR (CI 95%); *p*-value
PASI75 w12	255/305 (83.61%)	247/301 (82.06%)	0.614	NA	355/430 (82.56%)	145/176 (82.39%)	0.775	NA
PASI90 w12	183/305 (60%)	202/301 (67.11%)	0.069	NS	273/430 (63.49%)	112/176 (63.64%)	0.973	NA
PASI100 w12	137/305 (44.92%)	161/301 (53.49%)	**0.035**	NS	209/430 (48.60%)	89/176 (50.57%)	0.661	NA
PASI ≤2 w12	215/305 (70.49%)	224/301 (74.42%)	0.279	NA	315/430 (73.26%)	124/176 (70.45%)	0.484	NA
PASI75 w24	276/296 (93.24%)	268/293 (91.47%)	0.417	NA	385/418 (92.11%)	159/171 (92.98%)	0.716	NA
PASI90 w24	238/296 (80.41%)	232/293 (79.18%)	0.711	NA	325/418 (77.75%)	145/171 (84.80%)	0.053	0.61 (0.38–0.99); 0.049
PASI100 w24	177/296 (59.80%)	189/293 (64.51%)	0.239	NA	255/418 (61%)	111/171 (64.91%)	0.375	NA
PASI ≤2 w24	272/296 (91.89%)	253/293 (86.35%)	**0.031**	1.91 (1.11–3.27); 0.020	375/418 (89.71%)	150/171 (87.72%)	0.480	NA
PASI75 w52	261/281 (92.88%)	249/272 (91.54%)	0.557	NA	360/392 (91.84%)	150/161 (93.17%)	0.595	NA
PASI90 w52	236/281 (83.99%)	222/272 (81.62%)	0.460	NA	317/392 (80.87%)	141/161 (87.58%)	0.057	NS
PASI100 w52	186/281 (66.19%)	192/272 (70.59%)	0.266	NA	261/392 (66.58%)	117/161 (68.42%)	0.162	NS
PASI ≤2 w52	256/281 (91.10%)	238/272 (87.50%)	0.170	NS	349/392 (89.03%)	145/161 (84.80%)	0.721	NA
PASI75 w104	147/154 (95.45%)	136/150 (90.67%)	0.100	NS	202/218 (92.66%)	81/86 (94.19%)	0.637	NA
PASI90 w104	133/154 (86.36%)	126/150 (84%)	0.562	NA	182/218 (83.49%)	77/86 (89.53%)	0.181	NS
PASI100 w104	101/154 (65.58%)	107/150 (71.33%)	0.281	NA	145/218 (66.51%)	63/86 (73.26%)	0.255	NA
PASI ≤2 w104	147/154 (95.45%)	135/150 (90%)	0.066	NS	201/218 (92.20%)	81/86 (94.19%)	0.548	NA
PASI75 w156	64/69 (92.75%)	46/46 (100%)	0.062	NS	79/82 (96.34%)	31/33 (93.94%)	0.568	NA
PASI90 w156	54/69 (78.26%)	42/46 (91.30%)	0.065	NS	67/82 (81.71%)	29/33 (87.88%)	0.420	NA
PASI100 w156	39/69 (56.52%)	36/46 (78.26%)	**0.016**	0.36 (0.15–0.84); 0.018	51/82 (62.20%)	24/33 (72.73%)	0.283	NA
PASI ≤2 w156	60/69 (86.96%)	45/46 (97.83%)	**0.043**	NS	75/82 (91.46%)	30/33 (90.91%)	0.924	NA

PASI, psoriasis area and severity index; OR, odds ratio; CI, confidence interval; PsA, psoriatic arthritis; CMD, cardio-metabolic disease; NA, not applicable (multivariate analysis was not performed for these variables, as the *p*-value at univariate analysis was ≥0.2); NS, not significant.

**Table 3:** Effectiveness of brodalumab according to BMI classes.

	BMI ≥30	25 ≤BMI <30	BMI <25	Univariate analysis *p*-value	Multivariate analysis OR (CI 95%); *p*-value
PASI75 w12	89/110 (80.91%)	217/265 (81.89%)	196/231 (84.85%)	0.573	NA
PASI90 w12	64/110 (58.18%)	172/265 (64.91%)	149/231 (64.50%)	0.434	NA
PASI100 w12	45/110 (40.91%)	133/265 (50.19%)	120/231 (51.95%)	0.148	NS
PASI ≤2 w12	83/110 (75.45%)	189/265 (71.32%)	167/231 (72.29%)	0.716	NA
PASI75 w24	96/109 (88.07%)	241/252 (95.63%)	207/228 (90.80%)	**0.024** ^a^ ^,^ ^b^	^b^2.25 (1.06–4.79); 0.035
					^a^1.27 (1.24–6.63); 0.014
PASI90 w24	81/109 (74.31%)	210/252 (83.33%)	179/228 (78.51%)	0.121	NS
PASI100 w24	58/109 (53.21%)	169/252 (67.06%)	139/228 (60.96%)	**0.040** ^a^	^a^1.72 (1.08–2.73); 0.022
PASI ≤2 w24	98/109 (89.91%)	229/252 (90.87%)	198/228 (86.84%)	0.352	NA
PASI75 w52	88/100 (88%)	224/240 (93.33%)	198/213 (92.96%)	0.217	NA
PASI90 w52	78/100 (78%)	201/240 (83.75%)	179/213 (84.04%)	0.368	NA
PASI100 w52	59/100 (59%)	169/240 (70.42%)	150/213 (70.42%)	0.085	^a^1.65 (1.01–2.69); 0.046
PASI ≤2 w52	88/100 (88%)	213/240 (88.75%)	193/213 (90.61%)	0.727	NA
PASI75 w104	45/49 (91.84%)	130/137 (94.89%)	108/118 (91.53%)	0.533	NA
PASI90 w104	40/49 (81.63%)	117/137 (85.40%)	102/118 (86.44%)	0.725	NA
PASI100 w104	29/49 (59.18%)	97/137 (70.80%)	82/118 (69.49%)	0.308	NA
PASI ≤2 w104	45/49 (91.84%)	128/137 (93.43%)	109/118 (92.37%)	0.914	NA
PASI75 w156	13/14 (92.86%)	53/55 (96.36%)	44/46 (95.65%)	0.848	NA
PASI90 w156	12/14 (85.71%)	45/55 (81.82%)	39/46 (84.78%)	0.897	NA
PASI100 w156	9/14 (64.29%)	38/55 (69.09%)	28/46 60.87(%)	0.686	NA
PASI ≤2 w156	13/14 (92.86%)	50/55 (90.91%)	42/46 (91.30%)	0.974	NA

BMI, body mass index; OR, odds ratio; CI, confidence interval; NA, not applicable (multivariate analysis was not performed for these variables, as the *p*-value at univariate analysis was ≥0.2); NS, not significant.

a Overweight versus obese patients.

b Overweight versus normal-weight patients.

**Figure 2:**
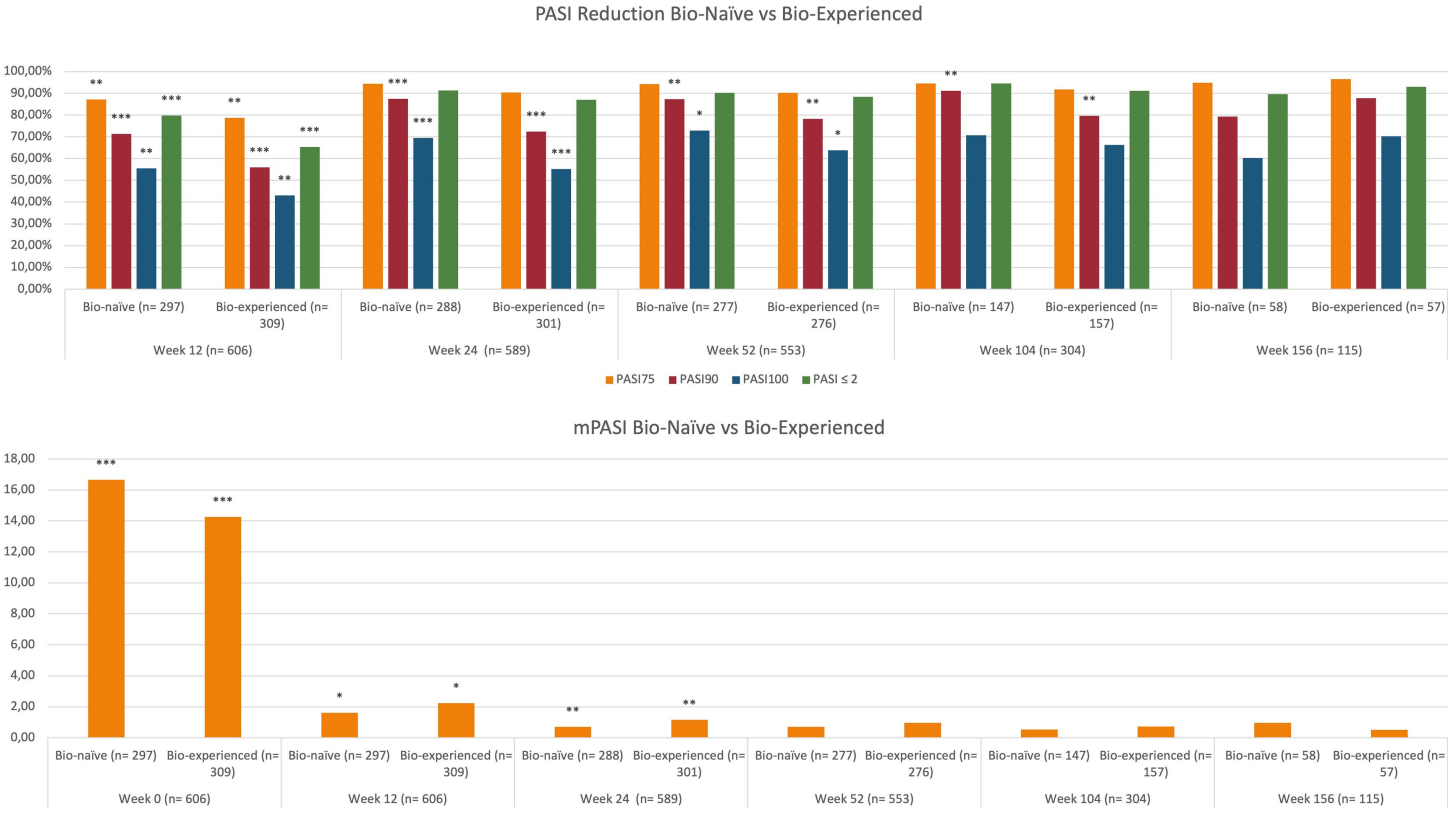
Effectiveness of brodalumab according to previous exposure to biological therapies. PASI, psoriasis area and severity index; mPASI: mean PASI. ^*^p-value <0.05; ^**^p-value ≤0.01; ^***^p-value ≤0.001.

At baseline, mean PASI was significantly higher in bio-naïve patients compared with bio-experienced (16.66 versus 14.26, *p* < 0.001). At week 12, we observed better responses regarding all effectiveness endpoints among the bio-naïve cohort. In particular, PASI 90 was achieved by 71.38% of bio-naïve compared with 55.99% of bio-experienced (*p* < 0.001) and PASI 100 by 55.56% versus 43.04% (*p* < 0.01). Higher rates of PASI 90 and PASI 100 were observed in bio-naïve patients also at weeks 24 and 52. At weeks 104 and 156, no significant differences were detected in relation to previous exposure to biologics. Complete univariate analysis and graphics regarding the effectiveness of brodalumab in bio-naïve and bio-naïve experiences are shown in Table 2 and Figure 2.

In our study, the concomitant diagnosis of PsA did not interfere with the effectiveness of brodalumab throughout the study period. The only exceptions are represented by a higher rate of PASI 100 response at week 12 (51.47% in patients without PsA and 40.77% in those with PsA; *p* < 0.05) and a higher proportion of patients without PsA who reached an absolute PASI of 2 or less at week 104 (98.67% versus 90.83%, *p* < 0.05).

The effectiveness of brodalumab was evaluated relative to BMI classes. Patients were defined as obese if they had a BMI ≥30, overweight if their BMI was ≥25 and lower than 30, and normal-weight patients if they had a BMI lower than 25. No significant differences were observed regarding the long-term effectiveness of brodalumab among the three groups in terms of mean PASI and percentage PASI reduction (Table 3). However, at week 24, PASI 100 was achieved by a significantly lower proportion of obese patients compared with overweight (53.21% versus 67.06%, *p* < 0.05).

Most of our patients (70.96%) had at least one difficult-to-treat area affected (scalp/face, palms/soles, nails and genitals). Brodalumab showed effectiveness in our population throughout the study period, regardless of the involvement of those body regions. No significant difference was detected regarding all effectiveness endpoints (Table 2).

We also evaluated the impact of CMD on treatment response to brodalumab. No significant differences were observed in the short- and mid-term (except for a higher PASI 100 in patients without CMD at week 12). While at week 104, all effectiveness endpoints were comparable between the groups, at week 156, we observed significantly better responses in patients without CMD (Table 2). In particular, PASI 100 was achieved by 56.52% of patients with concomitant CMD (versus 78.26%, *p* < 0.05) and PASI ≤2 by 86.96% (versus 97.83%, *p* < 0.05). Mean PASI was also significantly lower at week 156 in patients without CMD (0.30 versus 1.04, *p* < 0.01). Complete data on the differences among all subgroups in terms of mean PASI are shown in Supplementary Table S1.

### 3.4. Multivariate analysis

On multivariate analysis, at week 12, patients naïve to biological treatments were significantly more likely to achieve better responses concerning all the endpoints (PASI 75/90/100 and PASI ≤2; Table 2). At week 24, once again, bio-naïve patients had a higher probability of reaching PASI 90 [OR 2.65 (95% CI 1.72–4.1), *p* < 0.001] and PASI 100 [OR 1.79 (95% CI 1.27–2.52), *p* = 0.001]. At the same time point, overweight patients were significantly more likely to achieve PASI 75 (compared with both obese and normal-weight patients) and PASI 100 (compared with obese) (Table 3). The presence of CMD was a predictor of better rates of PASI ≤2 at week 24 [OR 1.91 (95% CI 1.11–3.27), *p* < 0.05]. At week 52, bio-naïve patients were significantly more likely to achieve both PASI 90 and 100 (Table 2). After 2 years of treatment, the only predictor of higher PASI 90 response was the bio-naïve status [OR 2.61 (95% CI 1.31–5.21), *p* < 0.01]. At week 156, the presence of CMD was a negative predictor of complete skin clearance [OR 0.36 (9%% 0.15–0.84), *p* < 0.05].

### 3.5. Drug survival and safety of brodalumab

**Figure 3:**
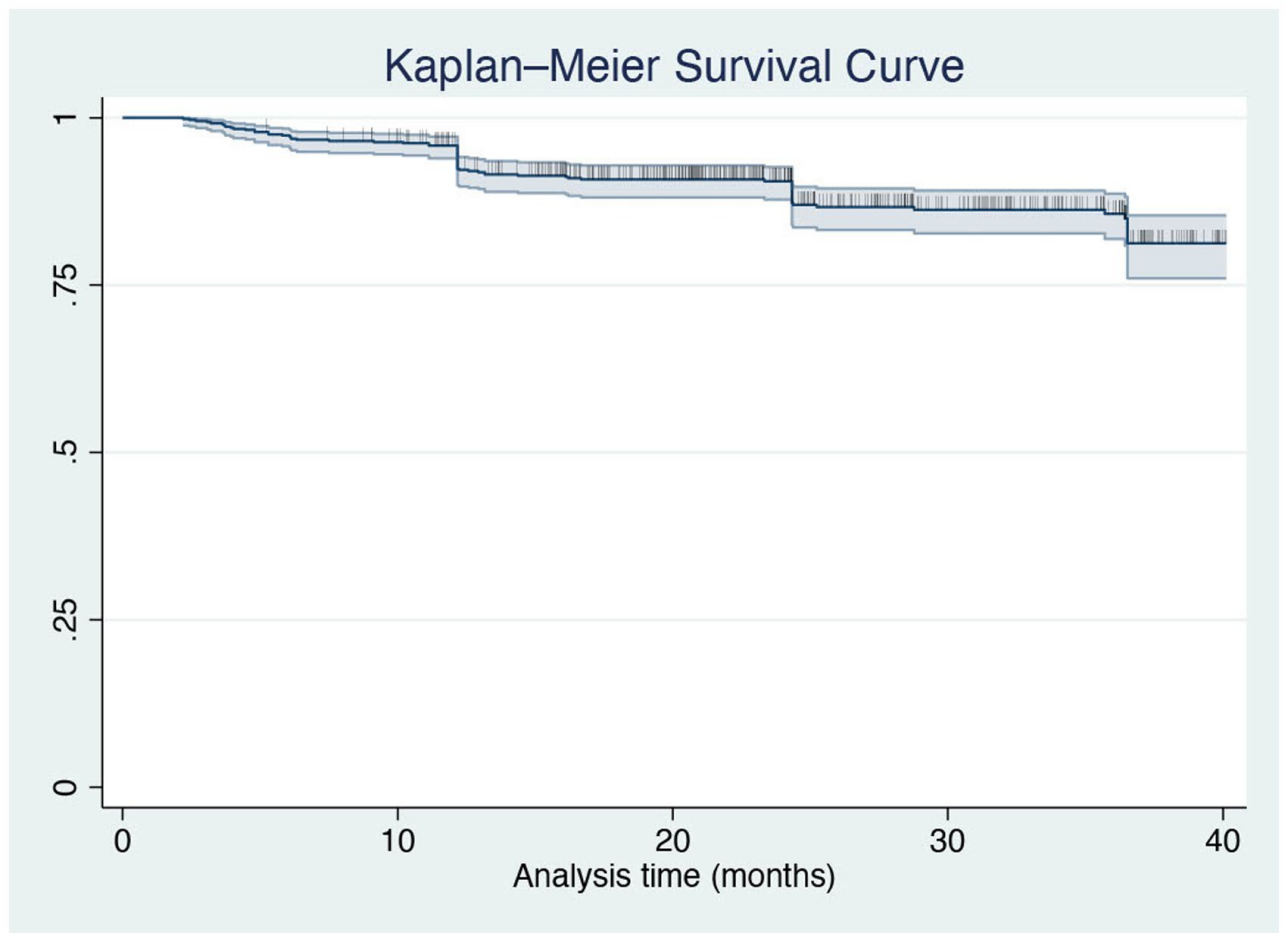
Kaplan–Meier curve of all-cause treatment discontinuation in all patients treated with brodalumab up to 36 months.

To assess the maintenance of brodalumab treatment, we evaluated the drug survival after 3 years using the Kaplan–Meier curve. At 36 months of therapy, 85.64% of our cohort was still on treatment with brodalumab (Figure 3). The log-rank test and Cox regression did not detect any differences in drug survival regarding BMI classes, comorbidities, involvement of difficult-to-treat areas and previous exposure to biologics. A total of 77 patients discontinued brodalumab during the study period. Thirty-nine patients (6.44%) experienced a loss of efficacy after the initial response to the treatment, while eleven (1.82%) were switched to other drugs because of primary inefficacy. Twenty patients (3.30%) experienced AEs leading to brodalumab discontinuation, including new-onset arthralgia (6 patients), persistent candidiasis (5), diarrhea (4), eczematous reactions (3). Furthermore, one patient developed a prostate cancer and one female received a diagnosis of breast cancer, both after 16 weeks of treatment with brodalumab. In agreement with the oncologists, they both stopped brodalumab to undergo surgery. Seven patients (1.16%) decided to stop the drug because of the persistence of complete clearance. The most commonly reported AEs were nasopharyngitis, headache, upper respiratory tract infections, *Candida* infections, arthralgia and diarrhea (Table 4). No cases of new-onset of inflammatory bowel diseases (IBD), suicidal attempts or severe COVID-19 infections were reported in our study. None of the eight patients with a diagnosis of viral hepatitis experienced reactivation.

**Table 4:** Safety profile of brodalumab throughout the study.

Adverse events	*N*. (% on total population)
Nasopharyngitis	28 (4.62%)
Headache	17 (2.81%)
Upper respiratory tract infections	15 (2.48%)
*Candida* infections	14 (2.31%)
Arthralgia	6 (0.99%)
Diarrhea	5 (0.83%)
Eczematous reaction	3 (0.50%)
Cancer diagnosis	2 (0.33%)
Total AEs	90 (14.85%)
AEs leading to discontinuation	**20 (3.30%)**

AE, adverse event.

## 4. Discussion

### 4.1. Comparison with clinical trials

Our study showed that the effectiveness of brodalumab is maintained over time, with one of the largest cohorts of patients to date completing two and three years of treatment. At week 12, we observed similar rates of PASI 75, 90 and PASI 100 responses, compared with Phase-3 clinical trials (83.3%, 70.3% and 41.9% in AMAGINE-1 study) (12–14). In our real-life experience, we observed better responses in the mid- and long-term, as 82.82% of our patients at week 52 reached PASI 90 (compared with 73%–75% of the three AMAGINE trials) and 68.35% achieved PASI 100 (versus 53%–56%). Our data on long-term effectiveness of brodalumab are also slightly better than those reported after 120 weeks from the clinical trial AMAGINE-2 (PASI 90: 75.6%, PASI 100: 61.1%) (19).

### 4.2. Comparison with other real-life studies

Compared with other real-life experiences, we observed similar data. Another Italian multicenter study (20) found at week 12 PASI 90 in 65% of patients and PASI 100 in 51.3%, higher than clinical trials and comparable with our study. At week 24, they reported PASI 90 and PASI 100 in 69.2 and 55.1%, respectively, which are lower than those observed in our study. Interestingly, the baseline characteristics of this cohort of patients were comparable with our population, as the mean BMI was 28.8%, and 47.4% were patients with previous exposure to biologics. In addition, short-term (12 and 24 weeks) PASI90 and PASI100 results are perfectly in line with another 24 weeks real-life study which observed PASI90 in 68.2% and PASI100 in 41.4% at week 12 and PASI90 in 75.6% and PASI100 in 60.9% at week 24, also involving a similar study population with almost 50% of bio-experienced patients (21). Real-life data on long-time exposure to brodalumab are currently limited (12–14). A recent study from Rompoti et al. (16) analyzed 91 patients with a lower baseline PASI compared to our population (10.3 versus 15.44), followed for 104 weeks. After 2 years of treatment, they found PASI 90 in 87.1% and PASI 100 in 80.7%. These data are higher than those observed in our experience and could be partially explained by lower disease severity at baseline.

In our study, brodalumab showed better clinical responses at week 12 in bio-naïve patients at the multivariate analysis, with similar outcomes after 3 years, compared with the bio-experienced subgroups. Similar data have also been observed regarding other biologics for treating psoriasis (22). Compared with another real-life experience from Mastorino et al. (14), we observed almost identical responses in terms of both PASI 90 (76% in bio-naive and 56% in bio-experienced) and PASI 100 (58% and 45%, respectively) at week 12. After 48 weeks of treatment, they did not report significant differences in PASI 100. However, PASI 90 responses were higher among bio-naïve patients (as observed in our multivariate analysis at the same time-point). Our findings were also consistent with those from Fargnoli et al. (18), who found that the bio-naïve status was a predictive factor of PASI 90 response at weeks 12 and 24. Regarding BMI, Mastorino et al. (14) also evaluated the effectiveness of brodalumab in obese and non-obese patients. At week 24, they found significantly higher responses in non-obese patients (PASI 100 in 75% of patients versus 45%). These data are consistent with our study, which found lower PASI 100 and PASI75 at week 24 in obese patients compared with overweight and normal-weight groups (14).

In our study, the analysis of the Kaplan Meier curves showed that 85.64% of our cohort was still receiving brodalumab after 3 years. Rompoti et al. (16) evaluated brodalumab drug survival after 104 weeks, reporting that 77.32% of the patients remained on treatment. Interestingly, in our experience, the drug survival was not impacted by initial treatment response, concomitant comorbidities, involvement of difficult-to-treat areas and bio-naïve or bio-experienced status, unlike other real-life studies.

### 4.3. Safety profile

Compared with phase-3 clinical trials and other real-life experiences, brodalumab did not show any new significant safety findings. Compared to clinical trials, we observed higher rates of *Candida* infections (11–13). Most of them resolved with topical antifungal therapy. Still, in five patients, the persistence of the symptoms required a switch to anti-IL-23 drugs, given the lower rates of *Candida* infection occurring with these drugs (23). The four patients who discontinued brodalumab because of persistent diarrhea underwent gastroenterological examination, and none received a IBD diagnosis. All AEs leading to brodalumab discontinuation resolved after drug withdrawal and/or switch to another biological drug. Moreover, none of our patients with a recent history of cancer experienced a relapse or a disease progression. In our experience, annual pulmonological visits and thoracic X-rays did not detect any sign of latent tuberculosis reactivation, as reported by Fowler et al. (24) in a review on anti-IL-17 drugs. Brodalumab was also well-tolerated by patients with a diagnosis of viral hepatitis, as repeated laboratory tests did not show transaminase elevations nor viral reactivation, consistent with data observed for other IL-17 and IL-23 inhibitors (25).

### 4.4. Limitations

The main limitations of our study are represented by its retrospective design and by the significant number of patients who were yet to attend the week 156 follow-up visit when the study was performed. Another limitation is the heterogeneity in clinical evaluations among dermatologists from different clinics. However, our experience represents the largest real-life to date on brodalumab, with more than six-hundred patients enrolled at baseline, with 115 patients completing at least 3 years of treatment.

## 5. Conclusion

Our study confirms the effectiveness of brodalumab in the short-term, showing that it is maintained over 3 years of continuous treatment, with comparable or better responses with respect to clinical trials. In our experience, bio-naïve patients showed better responses at weeks 12, 24 and 52, while the concomitant presence of CMD, regardless of BMI classes, was a negative predictor of effectiveness at week 156. Brodalumab showed a slower action in obese patients, but no significant differences were observed in the mid- and long-term among the three BMI classes. The involvement of difficult-to-treat areas and the concomitant diagnosis of PsA did not impact brodalumab effectiveness at any time, confirming this drug’s role in treating a heterogeneous real-life cohort of patients. No significant safety findings emerged in our 3 years study. The drug survival of brodalumab, in our experience, was not influenced by any patient characteristics. Further experiences should be reported to deeply analyse brodalumab effectiveness according to different parameters and its drug survival in a real-world setting.

## Data availability statement

The raw data supporting the conclusions of this article will be made available by the authors, without undue reservation.

## Ethics statement

Ethical review and approval was not required for the study on human participants in accordance with the local legislation and institutional requirements. Written informed consent for participation was not required for this study in accordance with the national legislation and the institutional requirements.

## Author contributions

LG and LI contributed on all stage of this study, conception and design of the study, performed the statistical analysis, manuscript revision, read, and approved the submitted version. AC, PM, and AN contributed to manuscript revision, read, and approved the submitted version. AB, FB, GA, GD, PD, CL, FL, and AM contributed to conception and design of the study, to data collection, and approved the submitted version. MB, CC, VD, GF, CF, FG, GG, CG, CL, MM, FS, and MT contributed to obtaining, analyzing and interpreting data, and approved the submitted version. All authors contributed to the article and approved the submitted version.

## Conflict of interest

The authors declare that the research was conducted in the absence of any commercial or financial relationships that could be construed as a potential conflict of interest.

## Publisher’s note

All claims expressed in this article are solely those of the authors and do not necessarily represent those of their affiliated organizations, or those of the publisher, the editors and the reviewers. Any product that may be evaluated in this article, or claim that may be made by its manufacturer, is not guaranteed or endorsed by the publisher.
