# Supplementary material for: Brodalumab for the treatment of plaque psoriasis in a real-life setting: a 3 years multicenter retrospective study—IL PSO (Italian landscape psoriasis)
Source: Front Med (Lausanne). 2023 Jul 3;10:1196966. doi: 10.3389/fmed.2023.1196966 (PMC10352451; doi:10.3389/fmed.2023.1196966)
Supplement: Supplementary file 1 [file Table_1.DOCX]

Supplementary Material

**Brodalumab for the treatment of plaque psoriasis in a real-life setting: a 3-year multicenter retrospective study - IL PSO (ITALIAN LANDSCAPE PSORIASIS)**

**Luigi Gargiulo, Luciano Ibba, Piergiorgio Malagoli, Fabrizio Amoruso, Giuseppe Argenziano, Anna Balato, Federico Bardazzi, Martina Burlando, Carlo Giovanni Carrera, Giovanni Damiani, Paolo Dapavo, Valentina Dini, Gabriella Fabbrocini, Chiara Franchi, Francesca Maria Gaiani, Giampiero Girolomoni, Claudio Guarneri, Claudia Lasagni, Francesco Loconsole, Angelo Valerio Marzano, Matteo Megna, Francesca Sampogna, Massimo Travaglini, Antonio Costanzo, Alessandra Narcisi^*^**

*** Correspondence:**

Alessandra Narcisi, MD, PhD

Email: alessandra.narcisi@humanitas.it

Dermatology Unit,

IRCCS Humanitas Research Hospital

20089, Rozzano (Milan)

Italy

# Supplementary Figures and Tables

## Supplementary Tables

**Supplementary Table 1.** Mean PASI at each time-point according to all analyzed variables. PASI: Psoriasis Area and Severity Index. mPASI: mean PASI; PsA: Psoriatic Arthritis; CMD: Cardio-Metabolic Disease; BMI: Body Mass Index.

|  | mPASI w0 | p-value | mPASI w12 | p-value | mPASI w24 | p-value | mPASI w52 | p-value | mPASI w104 | p-value | mPASIw156 | p-value |
| --- | --- | --- | --- | --- | --- | --- | --- | --- | --- | --- | --- | --- |
| Bio-naïve | 16.66 | **< 0.001** | 1.61 | **0.025** | 0.71 | **0.004** | 0.71 | 0.140 | 0.53 | 0.175 | 0.97 | 0.101 |
| Bio-experienced | 14.26 |  | 2.21 |  | 1.16 |  | 0.96 |  | 0.73 |  | 0.52 |  |
| PsA | 15.16 | 0.627 | 2.13 | 0.462 | 1.06 | 0.446 | 0.65 | 0.245 | 0.51 | 0.378 | 0.63 | 0.612 |
| No PsA | 15.51 |  | 1.88 |  | 0.91 |  | 0.89 |  | 0.67 |  | 0.79 |  |
| CMD | 15.55 | 0.708 | 1.98 | 0.704 | 0.93 | 0.822 | 0.78 | 0.540 | 0.60 | 0.683 | 1.04 | **0.008** |
| No CMD | 15.33 |  | 1.88 |  | 0.96 |  | 0.89 |  | 0.66 |  | 0.30 |  |
| Difficult Areas | 15.41 | 0.887 | 1.88 | 0.575 | 0.90 | 0.387 | 0.80 | 0.461 | 0.63 | 0.984 | 0.71 | 0.730 |
| No Difficult Areas | 15.50 |  | 2.05 |  | 1.05 |  | 0.93 |  | 0.63 |  | 0.82 |  |
| BMI ≥ 30 | 15.39 | 0.968 | 1.97 | 0.564 | 1.17 | 0.133 | 1.03 | 0.404 | 0.76 | 0.529 | 0.64 | 0.829 |
| 25 ≤ BMI <30 | 15.37 |  | 2.08 |  | 0.77 |  | 0.87 |  | 0.54 |  | 0.74 |  |
| BMI <25 | 15.53 |  | 1.74 |  | 1.02 |  | 0.71 |  | 0.68 |  | 0.78 |  |
